# Effects of water, sanitation and hygiene (WASH) education on childhood intestinal parasitic infections in rural Dembiya, northwest Ethiopia: an uncontrolled before-and-after intervention study

**DOI:** 10.1186/s12199-019-0774-z

**Published:** 2019-03-09

**Authors:** Zemichael Gizaw, Ayenew Addisu, Henok Dagne

**Affiliations:** 1 Department of Environmental and Occupational Health and Safety, Institute of Public Health, College of Medicine and Health Sciences, University of Gondar, Gondar, Ethiopia; 2 Department of Parasitology, School of Biomedical Science, College of Medicine and Health Sciences, University of Gondar, Gondar, Ethiopia

**Keywords:** Intestinal parasitic infections, WASH education, Children aged 6–59 months, Uncontrolled before and after intervention study, Rural Dembiya

## Abstract

**Background:**

Soil-transmitted helminthes (STH) infections are among the most common infections worldwide and affect the most deprived communities. Adequate water, sanitation, and hygiene (WASH) prevents environmental contamination, thereby preventing transmission of STH. Cognizant of this, WASH education was implemented in rural Dembiya to reduce intestinal parasitic infections. This study was, therefore, conducted to assess the impacts of the intervention on households’ WASH conditions and prevalence of intestinal parasitic infections.

**Method:**

An uncontrolled before-and-after intervention study was used. Cross-sectional studies were done before and after the intervention. Two hundred twenty-five and 302 under five children were recruited randomly at the baseline and endline, respectively. Data were collected using a structured questionnaire and observational checklists. Direct stool examination and Kato-Katz methods were used to identify parasites in the stool. We used percent point change and prevalence ratio (PR) to see the effects of the intervention on WASH conditions and prevalence of intestinal parasitic infections respectively. Pearson chi-squared and Fisher’s exact tests were used to test for statistically significant percentage point changes of WASH conditions. The effect of the intervention on intestinal parasitic infections was statistically tested on the basis of PR with 95% confidence interval (CI).

**Results:**

The baseline prevalence of intestinal parasitic infections was 25.8%, and the endline prevalence was 23.8%. The prevalence of intestinal parasitic infections was not significantly decreased at the endline compared with the baseline [PR = 0.92, 95% CI = (0.62, 1.38)]. *Ascaris Lumbricoides* was the most prevalent parasitic infection both at the baseline and endline. The proportion of children who had good hygienic condition increased from 1.3% at the baseline to 34.4% at the end line (*p* <  0.05). The percentage of mothers/care givers who washed hands at different pick times was significantly increased from 24.4% at the baseline to 68.2% at the endline (*p* <  0.001). The proportion of households who practiced home-based water treatment was significantly increased from 7.6% at the baseline to 47% at the endline (*p* <  0.001). The proportion of households who used sanitary latrine was increased from 32% at the baseline to 49% at the endline (*p* <  0.05).

**Conclusion:**

This before-and-after intervention study found that households’ WASH performance was significantly improved at the endline compared with the baseline. The endline prevalence of intestinal parasitic infections was slightly lower than the baseline prevalence; however, the reduction was not statistically significant. The local health office needs to strengthen the WASH education program, mobilize the community to construct WASH facilities, and support the community to sustain households’ WASH performance.

## Background

Soil-transmitted helminthes (STH) infections are among the most common infections worldwide and affect the poorest and most deprived communities. They are transmitted via eggs present in human feces, which contaminate soil in areas where sanitation is poor. The most common STH infections are roundworm (*Ascaris lumbricoides*), whipworm (*Trichuris trichiura*), and human hookworm (*Necator americanus* and *Ancylostoma duodenale*) [1–5].

The global health impact of STH infections was ranging between 4 million and 39 million disability adjusted life years (DALYs) [6]. In 2010, at least 1.3 billion people were estimated to be infected with at least one STH species [7]. The majority of the disease burden associated to STH infections is understood to be in children [8, 9] where infections are acquired through playing with contaminated soil and pica habits [10, 11]. In 2010, World Health Organization (WHO) estimated that 875 million children needed regular STH treatment [12]. STH infections in children can lead to under nutrition and growth faltering [7, 13–15], impair cognitive development [16–19], and cause anemia [20–23].

Intestinal parasitic worms enter the human host either through penetration of the skin or ingestion from contaminated hands or agricultural products [24–26]. Adequate sanitation prevents release of feces into the environment, thereby preventing transmission. Any single or combined water, sanitation, and hygiene (WASH) intervention reduces the risk of STH infections. A systematic review for the effect of latrine availability and utilization on STH infections found that latrine utilization reduced the risk of combined STH infection by about 50% [27]. A more recent review considered all WASH interventions found that WASH access and practices were generally associated with reduced odds of STH infection [28]. Studies provided strong evidence linking hygiene practices especially hand washing with reductions in STH infection [29–32]. WASH education is also one of the effective interventions to prevent and control transmission of intestinal parasitic infections [30, 31, 33, 34]. Cognizant of this, WASH education was implemented in rural Dembiya to reduce the prevalence of intestinal parasitic infections. The project was so called Dembiya NTD-WASH project. This study was, therefore, conducted to assess the impacts of the intervention on WASH and intestinal worms.

## Method

An uncontrolled before and after intervention study was used to determine the effect of WASH education on prevalence of childhood intestinal parasitic infections. Cross-sectional studies were done before and after the intervention. The study setting, study population, sample size determination and sampling procedures, data collection procedures, measurement of study variables, and stool examination methods were presented in a baseline study which is published elsewhere [35]. The baseline data were collected in May 2017, and the endline data were collected in May 2018. The sample size was re-calculated for the endline survey considering the prevalence of parasitic infections reported in the baseline [35].

### Description of the intervention

WASH education was given to the communities of five rural *kebels* (the lowest administrative unit in Ethiopia) and five rural schools. From five to six subgroups were formed in every *kebele* and school to make the health education interactive and manageable. We used school teachers, school children, university students, religious leaders, and health extension workers as WASH educators. The education was given every week for  three months immediately after the baseline survey and every week for  three months before the endline survey. Personal hygiene, effective hand washing practice, drinking water quality measures, food safety measures, waste management, health consequences of poor WASH, mode of transmission of intestinal parasitic infections, and prevention and control measures of intestinal parasitic infections were the content of the education. Role-play or drama, demonstration, group discussion, song, card-game, question and answer, and lecturing were the methodology we used. A total of 906 school children (399 females and 507 males) and 670 household heads participated in the five schools and *kebeles* in every week sessions.

In addition to health education, hand washing facilities from locally available materials were constructed in all schools. In every school, we put two to four jars with 25 l capacity and one to two plastic barrels with a capacity of 400 l to promote hand washing after visiting toilet. Hence, water is not available in all schools; students filled the containers every morning by shift. Environmental health clubs and school principals facilitated this task. We also prepared leaflets and posters to promote WASH. Leaflets were dispatched to school children and communities. The leaflets were more of pictorial to entertain illiterate individuals. What makes the living environment unhygienic, health consequences of unhygienic environment, and strategies to make the environment hygienic were the contents of the posters and leaflets.

**Fig. 1:**
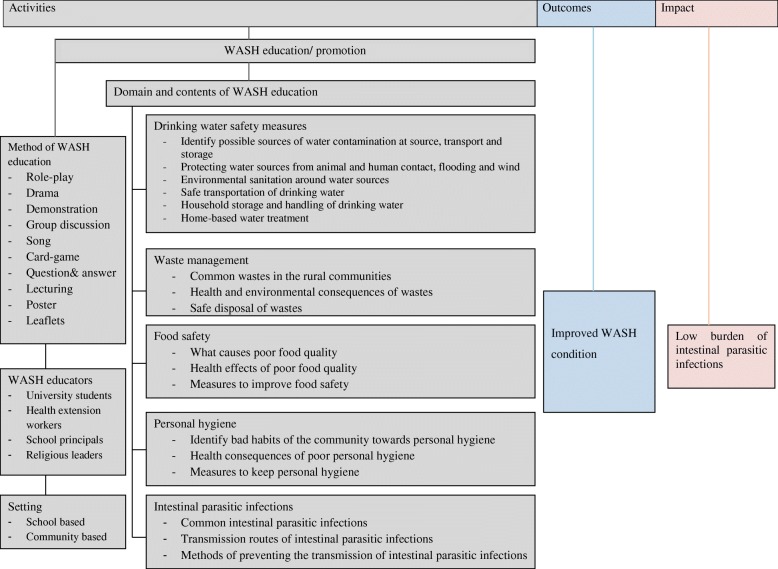
A frame work shows short summary of WASH intervention program in rural Dembiya, northwest Ethiopia

Weekly hygiene and sanitation supervision program was the other intervention we practiced. Home room teaches audit the hygiene condition of their students once a week and gave feedback to both the students and their families. Science teachers and environmental health clubs supervised the sanitation condition of the school environment every week and conducted sanitary campaign when necessary. We also established WASH committee in every *kebeles*. The committee comprised health extension worker, school principal, religious leaders, and *kebeles* administrator. The committee provided WASH education to the rural communities and supervised the hygiene and sanitation performances of the communities. Moreover, the committee mobilized the community and transferred health messages in public gatherings like community meeting and in churches (Fig. 1).

### Indicators tracked

Drinking water safety measures, environmental sanitation, personal hygiene, and parasitic infections were the four domains we used to track the change due to the interventions. Table 1 shows the household-level indicators tracked by the study. The detail of the indicators is well explained elsewhere [35].

**Table 1:** Household-level indicators by domain used to track changes due to the interventions in rural Dembiya, northwest Ethiopia

Domains	Indicators
Drinking water safety	% of households with children under five that practiced one or more home-based water treatment methods
Sanitation	% of households with children under five using hygienic latrine facilities
Hygiene	% of children whose personal hygiene condition is clean
	% of mothers or care givers whose hand washing practice is good
Parasitic infections	% of under five children who had one or more intestinal parasitic infections

### Statistical analysis

Data were entered using EPI-INFO version 3.5.3 statistical package. Data were imported, merged (baseline and endline data sets), and analyzed in SPSS statistical software package version 20. Composite variables were constructed by summing or scoring data collected in both rounds. The detail of scoring is published elsewhere [35]. For most variables, data were presented by frequency and percentage. The level of WASH conditions and prevalence of intestinal parasitic infections at the baseline and endline were compared. Percent point change with corresponding *p* value and PR with 95% CI were used to see the effects of the intervention on WASH conditions and intestinal parasitic infections respectively. Pearson chi-squared test was used to test for statistically significant changes between WASH indicators at baseline and endline. Fisher’s exact test was used for tables with expected values less than 5. Indicators with *p* values less than 0.05 and a 95% CI of PR not containing 1 showed a statistically significant change between baseline and endline.

### Role of the funding source

The funding organization of the study had no role in study design, data collection, data analysis, data interpretation, or writing of the report. The corresponding author had full access to all the data in the study and had final responsibility for the decision to submit for publication.

## Results

### Socio-demographic characteristics of study participants

Two hundred twenty-five and 302 children aged 6–59 months participated at the baseline and endline surveys, respectively. The majority of study participants were aged above 24 months at both surveys. The highest proportions (80% at the base line and 82.1% at the endline) of caregivers were illiterate (Table 2).

**Table 2:** Socio-demographic characteristics of study participants in the baseline (May 2017) and endline (May 2018) surveys in rural Dembiya, northwest Ethiopia

Socio-demographic variables	Baseline survey	Endline survey
	*n* (%)	*n* (%)
Sex of children		
Male	106 (47.1)	158 (52.3)
Female	119 (52.9)	144 (47.7)
Age of children		
6–24	59 (26.2)	96 (31.8)
> 24	166 (73.8)	206 (68.2)
Maternal education		
No formal education	180 (80.0)	248 (82.1)
Have formal education	45 (20.0)	54 (17.9)

### WASH conditions

At baseline, the general hygienic condition of 1.3% of the children was good. Conversely, the percentage of children that had good hygienic condition increased to 34.4% at the endline (*p* <  0.05). The percentage of mothers/care givers who washed hands after visiting toilet or changing baby’s diaper or touching wastes, before eating and food preparation, was significantly increased from 24.4% at the baseline to 68.2% at the endline (*p* <  0.001). The proportion of households who used sanitary latrine was significantly increased from 32% at the baseline to 49% at the endline (*p* <  0.05). The proportion of households who practiced home-based water treatment was significantly increased from 7.6% at the baseline to 47% at the endline (*p* <  0.001) (Table 3). Water guard was the commonest home-based water treatment method practiced by the rural communities both at the baseline and endline (Table 4).

**Table 3:** WASH condition before (May 2017) and after the intervention (May 2018) in rural Dembiya, northwest Ethiopia

WASH variables	Baseline	Endline	% point change	*p* value
	*n* (%)	*n* (%)		
Percentage of children whose personal hygiene condition is clean	3 (1.3)	104 (34.4)	33.1	< 0.05*
Percentage of mothers or caregivers whose hand washing practice is good	55 (24.4)	206 (68.2)	43.8	< 0.001
Percentage of households practiced home-based water treatment	17 (7.6)	142 (47.0)	39.4	< 0.001
Percentage of households used sanitary latrine	72 (32.0)	148 (49.0)	17.0	< 0.05

*Fisher’s exact test

**Table 4:** Common home-based water treatment methods practiced before (May 2017) and after the intervention (May 2018) by the community of rural Dembiya

Home-based water treatment methods	Baseline (*n* = 225)	Endline (*n* = 302)
	*n* (%)	*n* (%)
Water guard (chlorine solution)	13 (5.8)	142 (47.0)
Solar disinfection	1 (0.4)	–
Boiling	2 (0.9)	–
Cloth sieve filtration	1 (0.4)	–
No treatment methods used	208 (92.4)	160 (53.0)

### Prevalence of intestinal parasitic infections

**Fig. 2:**
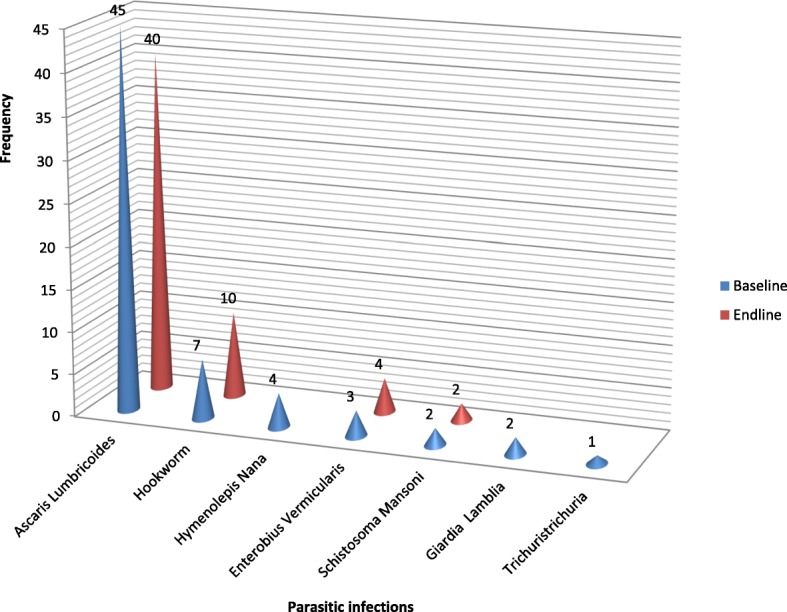
Common intestinal parasitic infections identified among children aged 6–59 months at the baseline (May 2017) and endline (May 2018) in rural Dembiya, northwest Ethiopia

From a total of 225 children included in the baseline survey, 58 of the children were infected with one or more intestinal parasitic infections. The prevalence of intestinal parasitic infections at the baseline was, therefore, found to be 25.8%. One hundred children were unable to give stool sample at the endline. As a result, stool sample was taken from 202 children at the endline. Forty-eight children out of 202 were infected with one or more intestinal parasitic infections at the endline. The endline prevalence was, therefore, found to be 23.8%. At the endline, four children had double and two had triple infections. *Ascaris Lumbricoides* was the most prevalent parasitic infection both at the baseline and endline (Fig. 2). The prevalence of intestinal parasitic infections was not significantly decreased at the endline compared with the baseline [PR = 0.92, 95% CI = (0.62, 1.38)] (Table 5).

**Table 5:** The effect of WASH education on childhood intestinal parasitic infections in rural Dembiya, northwest Ethiopia

Intestinal parasitic infections	Baseline (*n* = 225)	Endline (*n* = 202)	PR (95% CI)
	*n* (%)	*n* (%)	
Percentage of under five children who had at least one intestinal parasitic infection	58 (25.8)	48 (23.8)	0.92 (0.62, 1.38)

## Discussion

The present study assessed the effects of WASH education on childhood parasitic infections in rural Dembiya, northwest Ethiopia. The prevalence of childhood intestinal parasitic infection was found 25.8% at the baseline and 23.8% at the endline. Children in the study area had developed one or more intestinal parasitic infections due to the fact that the population in the area had poor access to sanitation. During June 2017, clean water and latrine coverage in the district was 26.6% and 55% respectively [36]. The findings of the current study are similar with the reports of studies in Wonji Shoa Sugar Estate (24.3%) [37] and in Butajira town (23.3%) [38]. The baseline and endline prevalence of intestinal parasitic infections reported by this study is also lower than the findings of studies in Wondo Genet (85.1%) [39] and Hawassa Zuria District (51.3%) [40]. The lower prevalence might be due to the fact that anthelmintic drugs were administered by nongovernmental organizations in the current study area before the commencement of this project.

This study revealed that WASH education was significantly associated with households’ sanitation performance. Water safety measures, children’s hygiene condition, mothers’/care givers’ hand washing practice, and latrine utilization were significantly improved at the endline compared with the baseline conditions. The effect of WASH education on households’ WASH performance might be due to the fact that health education increases awareness on good WASH practices and encourages behavioral change [41–44]. The finding of this study is in line with intervention-based studies in Mali and India [45, 46]. However, the interventions in Mali and India were more effective than our intervention to promote households’ WASH performance due to the fact that the interventions in Mali and India were community-based total sanitation (CBTS) or community leading total sanitation (CLTS). CBTS or CLTS is one of the effective WASH promotion approaches to empower rural communities and to develop community ownership for better behavioral change [47–50].

Although it is not statistically significant, this intervention-based study reported that the prevalence of intestinal parasitic infections was slightly reduced at the endline compared with the baseline. This slight reduction might be due to the fact that WASH interventions have no curative effect once children are infected to the parasites. However, other similar studies reported significant reduction of intestinal parasitic infection due to WASH interventions [34, 45, 46]. This might be due to the fact that health education increases awareness about the potential health impacts of poor environmental sanitation and motivates the communities to take care for their health so that households implement barriers to prevent occurrence of infections [30, 31, 33, 51].

Lastly as a limitation, this research was uncontrolled before and after intervention study with no control group. The evidence generated may not be strong because of the weak nature of the research design. We could not clearly know whether our intervention or other activities brought WASH improvement and in the study area because of the absence of control group. We recommend randomized controlled trial studies in the area to generate concrete evidence on the link between WASH interventions and intestinal parasitic infections in the rural setups.

## Conclusion

This before and after intervention study found that households’ WASH performance was significantly improved at the endline compared with the baseline. The endline prevalence of intestinal parasitic infections was slightly lower than the baseline prevalence; however, the reduction was not statistically significant. The local health office needs to strengthen the WASH education program, mobilize the community to construct WASH facilities, and support the community to sustain households’ WASH performance.

## Abbreviations

CBTS: Community-based total sanitation
CI: Confidence interval
CLTS: Community leading total sanitation
DALYs: Disability adjusted life years
NALA: Neglected tropical diseases advocacy learning action
NTD: Neglected tropical diseases
PR: Prevalence ratio
SPSS: Statistical package for social sciences
STH: Soil-transmitted helminthes
WASH: Water, sanitation, and hygiene
WHO: World Health Organization
